# Integrating mental health support into HIV prevention: An organizational framework for NGOs, informed by Ukraine’s experience

**DOI:** 10.1371/journal.pmen.0000516

**Published:** 2025-12-30

**Authors:** Vitalii Klymchuk, Olha Petrash, Oleksandra Vynogradova, Viktoriia Gorbunova, Volodymyr Kurpita, Shirley Ko

**Affiliations:** 1 Department of Social and Applied Psychology, Zhytomyr Ivan Franko State University, Zhytomyr, Ukraine; 2 Department of Social Sciences, University of Luxembourg, Esch-sur-Alzette, Luxembourg; 3 Community Action for HIV Control, Pact Ukraine, Kyiv, Ukraine; 4 Prevention and Demand Generation, FHI 360 Ukraine, Kyiv, Ukraine; 5 Department of Behavioural and Cognitive Sciences, University of Luxembourg, Esch-sur-Alzette, Luxembourg; 6 Global Health, Pact, Washington, District of Columbia, United States of America; PLOS: Public Library of Science, UNITED KINGDOM OF GREAT BRITAIN AND NORTHERN IRELAND

## Abstract

The objective was develop and promote an organizational policy framework that enables non-governmental organisations (NGOs) involved in HIV prevention in Ukraine to systematically integrate mental health and psychosocial support (MHPSS) into their operations, thereby improving both client and staff well-being and strengthening HIV prevention outcomes. A practice-based, participatory policy development and implementation process was launched, embedded within the “Community Action for HIV Control” (CAHC) project, encompassing 14 regional NGOs in Ukraine, incorporating stakeholder consultations, policy co-creation, and training. An iterative co-development of a mental health organizational framework informed by global best practices and local feasibility, with input from participating NGOs, was implemented, based on the previous situational analysis of NGO mental health capacities and practices and implementation of the development and dissemination of a training-of-trainers programme for non-mental health professionals, delivery of evidence-based psychosocial interventions and six months of supervision. All 14 participating NGOs adopted the organizational framework, which defines four key domains: planning and resource allocation, staff support, client mental health services (via a stepped care model), and community engagement. NGOs integrated various levels of MHPSS into existing HIV services, supported by structured protocols, referral directories, and internal monitoring mechanisms. Challenges identified included funding constraints, stigma, limited specialist availability, and staff workload concerns. Recommendations addressed sustainability through task-sharing, partnerships, internal capacity building, and anti-stigma initiatives. Integrating mental health into HIV prevention services through a structured organizational framework was found both feasible and essential, even in resource-limited settings. The framework offers a scalable model for other NGOs globally and reinforces the principle that mental health is a vital component of comprehensive HIV prevention. Sustainable implementation requires organisational commitment, staff support, community linkages, and continuous capacity strengthening.

## Introduction

Integrating mental health into HIV prevention and care is increasingly recognized as essential for effective HIV response [1–3]. People at risk of HIV and those living with HIV experience significantly higher rates of mental health conditions compared to the general population [4]. Depression, anxiety, substance use, and trauma-related conditions are prevalent among key populations (such as LGBTQ+ individuals, people who inject drugs, and sex workers) due to stigma, discrimination, and psychosocial stressors. These challenges can increase risk behaviors and hinder engagement in HIV prevention and treatment programs [5,6]. Consequently, unmet mental health needs represent a barrier to achieving HIV targets and ending the epidemic.

In response, global health authorities have called for an integrated approach to HIV and mental health care. The World Health Organization (WHO) and UNAIDS emphasize that HIV prevention, testing, treatment, and care services should incorporate mental health support as one of the core components [2]. Integrating mental health services into HIV programs not only improves HIV-related outcomes (such as retention in care and viral suppression) but also expands access to mental health care for underserved groups. Evidence from research indicates that combined HIV and mental health interventions can reduce depression and substance use, improve mood and social functioning, and even lower stigma among people living with HIV [6,7]. Frontline providers in integrated programs report greater comfort in addressing mental health issues, and clients are more likely to accept referrals for needed mental health services [3].

In Ukraine, HIV-focused NGOs are often at the forefront of reaching vulnerable communities and well-positioned to deliver or facilitate mental health support. However, many NGOs lack a formal framework or dedicated resources to address mental health, resulting in inconsistent quality of care and fragmented, donor-dependent efforts. Delivery of mental health services may be confined to specific NGO departments or teams within short-lived projects, limiting their impact on meeting the mental health needs of HIV-vulnerable populations and on providing support to NGO staff. Establishing a clear organizational approach to mental health provides a critical opportunity for NGOs to systematically and sustainably incorporate psychosocial support across all levels of the organization.

As was revealed in a recent study, only 53% of such NGOs reported providing mental health services as a durable aspect of programming; 60% offered such services primarily through project-based funding [8]. Only 47% screened clients used standardized measures (e.g., PHQ-9, GAD-7), and only 20% had a formal mental health policy. However, most NGOs reported a high perceived need and motivation to improve mental health services. Based on the study results, several recommendations for the NGOs were proposed, pointing out the need for developing and adopting formal mental health organizational frameworks, enhancing training and capacity building, securing sustainable funding, building robust partnerships and referral networks, standardizing screening and monitoring, promoting shared learning and best practices.

This paper provides an overview of the mental health organizational framework, drawing on recently developed policy documents and technical guidelines for integrating mental health into the work of NGOs active in HIV prevention. It offers recommendations to ensure mental health becomes an integral, sustainable element of NGO-led HIV prevention programs.

## Background

### Best practices for integrating mental health in the work of NGOs involved in HIV prevention

Effective integration of mental health into HIV prevention work requires a multifaceted approach. Several global best practices shaped the development of a mental health organizational framework for HIV-focused organizations in Ukraine and also hold relevance for similar organizations worldwide: Routine Screening and Referral [1,9], Task Sharing and Stepped Care [10–12], Psychoeducation and Prevention [2], Linkages and Holistic Care [13,14], Staff Training and Support [15–17],Anti-Stigma and Rights-Based Approach [18–20]. Adhering to these best practices can help NGOs create an enabling environment for mental health integration. These approaches are reflected in the mental health policy framework discussed below, which translates the best practices into concrete organizational policies and procedures.

### Piloting the Integration of mental health in the work of Ukrainian NGOs involved in the HIV prevention

The pilot project integrating mental health support into the work of NGOs involved in HIV prevention was conducted from March to December 2024 within the framework of the broader CAHC project, which was implemented in Ukraine by Pact with support from the U.S. Agency for International Development. The goal of this initiative was to accelerate Ukraine’s effort to achieve epidemic control by 2030 through improved prevention, testing, and linkage to care among key and priority populations.

This pilot project aimed to enhance access to evidence-based mental health support for key and priority populations, including people living with HIV. It focused on implementing short-term, scalable psychosocial interventions disseminated by a structured Training of Trainers program and delivered by non-mental health professionals. The initiative involved training NGO service providers, offering six months of supervision, and integrating mental health support into routine service delivery. Key project components included situational analysis, training in scalable psychosocial interventions, knowledge transfer, piloting mental health support delivery, and co-development and approval of a mental health organizational framework.

Detailed information about the project elements is available in S1 File. Overall, 17 NGOs involved in HIV prevention from all regions of Ukraine were invited to participate in the project, of them 14 committed, including: Zakarpattia, Lviv and Ivano-Frankivsk oblasts (West), Zhytomyr, Chernihiv oblasts (North), Kyiv, Poltava, Cherkasy and Kirovograd oblasts (Centre), Dnipropetrovsk, Donetsk (East), Zaporizhzhia, Mykolaiv and Odesa oblasts (South). From May to December 2024, 292 non-mental health professionals from these NGOs were trained in delivering mental health support. During this same period, 361 NGO staff and 5,541 NGO clients received direct mental health support.

The framework development process consisted of several key steps. We began with initial consultations with NGO representatives regarding the need and potential elements of such a framework, followed by expert analysis of the existing Health Assessment Europe`s mental health services quality criteria, previously adapted and piloted in Ukraine [21]. From this analysis, the criteria that were most feasible for the NGOs to implement and aligned with their expectations were jointly reviewed. Next, we developed a draft framework, which was shared with NGOs bilaterally and via group consultations for their feedback. Once finalized, each NGO adapted and adopted the framework, making specific context-specific adjustments to ensure relevance and applicability.

All participating NGOs were involved in the framework development process; in September-November 2024, they finalized a shared blueprint, and in December 2024, each organization adopted the framework tailored to their organizational capacities and culture.

The research team adhered to the Declaration of Helsinki and the ethical regulations of the National Psychological Association of Ukraine. The study protocol, survey, and informed consent forms were approved by the Ethics Committee of the Zhytomyr Ivan Franko State University, approval №02–0709/2024, 17 January 2024.

### Organizational framework for mental health integration

The mental health organizational framework developed for and with the HIV-focused NGOs provides a structured pathway to embed psychosocial support within the organization’s mission and operations. At its core, the framework declares mental health as a fundamental organizational value – the well-being of clients and staff is vital to the NGO’s success. Mental health considerations are meant to *“permeate all organizational processes,”* affecting all services and activities, and the policy is designed to integrate with existing organizational policies (such as those on human resources, safeguarding, or client services) following the “mental health in all policies” approach *(EC, 2017)* [22].

### Mental health organizational framework structure and key components

**Fig 1:**
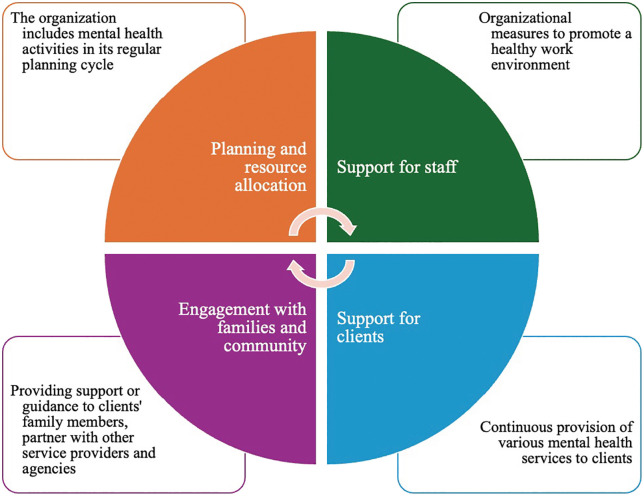
Elements of the mental health organizational framework.

The mental health organizational framework delineates specific domains of action within the NGO: (1) planning and resource allocation, (2) support for staff mental health, (3) support for client mental health, and (4) engagement with families and the broader community (Fig 1)

### Planning and resources

The framework mandates that the organization include mental health activities in its regular planning cycle. Concretely, the NGO should develop an annual Mental Health Action Plan that identifies objectives, activities, responsible persons, timelines, and indicators related to mental health integration. This plan must be reviewed yearly and informed by an assessment of needs and the results of past activities. For example, if 30% of clients used counseling services in the past year, the plan might aim to increase outreach or capacity accordingly. The policy also encourages budgeting for mental health, seeking dedicated funding, or allocating a portion of the NGO’s own funds to sustain psychosocial support services. In essence, mental health should be incorporated into project proposals and internal budgets. The planning component ensures leadership commitment and resource mobilization for implementation.

### Support for staff mental health

The framework recognizes that staff are the NGO’s most important resource, as their mental health has a direct impact on the service quality, and peer-workers share the same vulnerabilities as the population they serve. It devotes a section to employee mental well-being and requires organizational measures to promote a healthy work environment. These include managing workloads fairly (to prevent excessive stress), ensuring transparent compensation, providing positive feedback and opportunities for professional growth, and preventing workplace issues like bullying or harassment. These measures address systemic factors that influence staff stress and morale. In addition, the policy outlines direct mental health support for staff.

All employees should benefit from mental health promotion activities (such as workshops on stress management or resilience) and anti-stigma education to encourage help-seeking. The NGO should offer or facilitate services for staff like those for clients: psychological first aid after critical incidents, psychoeducation, voluntary screening/assessment, short-term counseling or low-intensity interventions, and referral to external psychotherapy or psychiatric care when needed. Notably, if a staff member has experienced mental illness, the organization will support their return to productive work (for instance, by adjusting duties during recovery).

These staff services must be provided anonymously or by external providers, as appropriate, to maintain confidentiality and trust. Furthermore, the framework calls for regular monitoring of staff well-being through anonymous surveys or standardized tools to gauge stress, fatigue, and burnout levels. The framework explicitly requires developing a protocol to respond to suicide attempts or the death of an employee by suicide to guide managers and colleagues in providing immediate support and postvention.

### Support for clients` mental health

The core of the framework focuses on integrating mental health services into the NGO’s client-facing programs. The organization is expected to continuously provide its clients with various mental health and psychosocial support services. According to the framework, these services include psychological first aid (for clients in acute distress or crisis), psychoeducation (providing information about mental health and coping strategies), low-intensity psychological interventions (e.g., basic counseling or support sessions focusing on problem-solving, which trained non-specialists can deliver), psychological counseling, psychotherapy and psychiatric treatment. The breadth of services offered may depend on the NGO’s resources and trained personnel, but even smaller organizations are encouraged to provide at least basic psychosocial support and referrals.

A crucial feature of the framework for client services is the **two-tier (stepped) care model**.

The first tier consists of NGO staff who are not mental health specialists, such as case managers, outreach workers, or peer navigators, who receive training to deliver basic mental health and psychosocial support support. These front-line workers can perform interventions like active listening, counseling, stress reduction techniques, and psychoeducation.

If a client’s condition does not improve despite receiving the first-tier intervention or the case is beyond the staff member’s expertise, the policy instructs staff to refer the client to the **second tier of care**. The second tier comprises mental health professionals (psychologists, psychotherapists, or psychiatrists) either on staff or contracted by the NGO. These specialists can provide more intensive services such as mental health assessment, psychotherapy or psychiatric evaluation.

Should a client require services beyond what the NGO and its consultants can offer (for example, psychiatric hospitalization, addiction rehabilitation, or specialized therapy), the NGO will refer the client to an external facility. A directory of partner organizations and public services lists up-to-date contacts and entry points for mental health care. After an external referral, the NGO staff should follow up (with the client’s consent) to check whether the client accessed the service and if their needs were met.

### Engagement with families and community

Beyond the organization’s direct services, the framework extends to family and community engagement, recognizing that their social context influences client well-being. The NGO commits to providing mental health support or guidance to clients’ family members, should they desire it. For example, family counseling sessions or psychoeducational materials might be offered to help families support their loved one’s mental health and adherence to HIV prevention or treatment.

The policy also encourages the NGO to actively participate in community initiatives to raise awareness about mental health and reduce stigma. These initiatives could include public events, social media information campaigns, collaboration with schools or local groups, and contributions to multi-stakeholder forums on mental health and HIV. By doing so, the NGO helps strengthen the community’s capacity to understand and address mental health needs, which benefits its client population and beyond.

Lastly, the framework calls for **routine evaluation of client experience** with mental health services. The NGO should gather feedback (anonymously when possible) from clients about the support they received, their satisfaction, and suggestions for improvement. This can be done through surveys after services or periodic feedback sessions, at least biannually.

In summary, the policy framework provides a comprehensive template for HIV prevention NGOs to integrate mental health into their operations. The following section discusses how NGOs can implement these policy elements in practice and measure their progress.

## Discussion: Challenges and recommendations

While implementing mental health integration in HIV prevention NGOs is beneficial, it is not without challenges. Anticipating these challenges and proactively addressing them is crucial for success. Below, we outline key obstacles and provide policy recommendations for sustainable implementation.

### Limited resources and funding

Many NGOs operate under tight budgets, and adding mental health services can strain financial and human resources.

#### Recommendation.

Mainstream mental health into existing programs to leverage current funding (e.g., training outreach workers rather than hiring all new staff). Seek dedicated funds by highlighting that mental health integration improves HIV outcomes – for example, use data to show that treating depression can improve HIV prevention adherence. Diversify funding by approaching donors interested in mental health or corporate social responsibility programs. In-kind partnerships can also help; local universities or clinics might provide low-cost trainee psychologists or counselors. Emphasize task-sharing to utilize resources and initiate low-cost, high-impact interventions efficiently.

#### Stigma.

Within some organizations or communities, mental health issues may not be fully understood or may carry stigma, leading to low prioritization.

#### Recommendation.

Incorporate stigma-reduction efforts as part of the policy rollout. Conduct awareness sessions for all NGO staff to underscore that mental health is a genuine health issue and integral to HIV work. Share success stories and testimonials to humanize mental health needs. It is also beneficial to have leadership champions – NGO directors or managers endorsing the policy and modeling openness (for instance, discussing stress management in staff meetings).

### Insufficient skilled personnel

NGOs may lack staff with mental health expertise, and hiring psychologists can be complex due to workforce shortages or cost.

#### Recommendation.

Utilize training and capacity building as outlined in the policy to grow skills internally. Identify staff interested in psychosocial support and invest in their development (workshops, online courses, mentorship). Partner with mental health professionals willing to volunteer or work part-time to supervise cases – for example, a pro bono arrangement with a local psychology association or tapping into diaspora experts via telemedicine. *N*etworks should be created with other NGOs to share a roving psychologist or conduct joint training, spreading the cost.

### Service integration and workload

Staff may feel that integrating new mental health tasks adds to their workload or distracts from core HIV activities.

#### Recommendation.

Emphasize that mental health integration enhances outcomes, not overburden staff. Through the policy’s organizational measures (fair workload distribution, hiring additional staff if needed, etc.), ensure that duties are balanced. Integrating services can also mean modifying workflows – for instance, combining mental health screening with existing intake forms. Hence, it becomes a routine part of an outreach visit rather than an extra separate task. Provide tools that make integration easier (simple screening checklists, referral cheat sheets) and allow staff to give feedback on what is working. By involving staff in designing the implementation, they are more likely to feel ownership rather than a burden.

### Coordination with external services

Successfully referring clients to external mental health or social services can be demanding if those services are scarce, far away, or of variable quality.

#### Recommendation.

Build strong referral partnerships as part of policy implementation. This could involve signing a Memorandum of Understanding with a local clinic or psychologist for priority referrals or establishing a feedback loop to inform the NGO about the outcomes of referrals. The policy suggests maintaining an up-to-date list of referral options – take this further by regularly meeting with those external providers to keep relationships warm. If services are far from clients, consider arranging transportation support or using tele-counseling options (some organizations set up tablets for clients to speak with a counselor remotely). In addressing these challenges, NGOs must remember that incremental progress is still valuable. The recommendations above can be implemented gradually – each step (like forming one partnership or training a handful of staff) will build momentum. Over time, overcoming these challenges will lead to a more resilient, capable organization and demonstrably better outcomes for the communities served.

## Conclusion

Mental health integration within HIV-focused NGOs is not just a complementary effort but a necessity for delivering comprehensive, person-centered care. As this paper outlines, unaddressed mental health needs can undermine HIV prevention and treatment gains, whereas providing psychosocial support can empower clients to adopt and sustain healthy behaviors. The proposed organizational framework for mental health offers NGOs a clear pathway to embed mental health and psychosocial support services into their operations, ensuring that the well-being of clients and staff is integrated into organizational practice and culture. By adhering to guiding principles, NGOs reinforce their commitment to human rights and service quality in the HIV response.

Implementing the policy will require thoughtful planning, resourcefulness, and collaboration. However, the benefits are substantial: clients receiving integrated care are more likely to engage in prevention programs, adhere to treatment, and experience improved quality of life, while staff working in a supportive environment are more productive and less prone to burnout. Early experiences from integrated programs and policies, summarized here, indicate that even in resource-limited settings, meaningful integration is achievable through task sharing, partnerships, and community engagement.

In conclusion, adopting a mental health policy is a forward-looking move for HIV-focused NGOs in Ukraine and beyond. It aligns with international recommendations and the realities of client needs. It operationalizes the concept that mental health is health and that no HIV prevention effort is complete without addressing psychological well-being. The hope is that with sustained commitment and adaptive implementation of this framework, NGOs will help close the mental health gap in HIV services, thereby enhancing the effectiveness and humanity of the global HIV response.

## Supporting information

S1 File ANNEX. Project activities: strengthening psychosocial support services for key and priority populations in Ukraine. (DOCX)
