# Supplementary material for: Integrating mental health support into HIV prevention: An organizational framework for NGOs, informed by Ukraine’s experience
Source: PLOS Ment Health. 2025 Dec 30;2(12):e0000516. doi: 10.1371/journal.pmen.0000516 (PMC12798232; doi:10.1371/journal.pmen.0000516)
Supplement: S1 File — (DOCX) [file pmen.0000516.s001.docx]

**ANNEX**

**Project Activities: Strengthening Psychosocial Support Services for Key and Priority Populations in Ukraine**

1. **Introduction**

This project aims to enhance access to evidence-based mental health support for key and priority populations, including people living with HIV (PLHIV), in Ukraine. By scaling up structured and manualized psychosocial support (PSS) approaches adapted to the Ukrainian context, the initiative focuses on empowering non-mental health professionals to deliver effective support services. The project integrates training, supervision, service provision, and monitoring mechanisms to ensure sustainable and high-quality implementation across non-governmental organizations (NGOs).

1. **Scope of Work**

The project involves multiple interrelated activities designed to establish a robust PSS intervention model at the community level. The key activities include:

**2.1 Selection of Evidence-Based Psychosocial Interventions**

- Identify and adapt a short-term, scalable, manualized PSS intervention suitable for non-mental health professionals.
- Ensure contextual appropriateness.

**2.2 Capacity Building for Service Providers**

- Identify and select a pool of certified trainers for the chosen intervention, with provisions for their supervision.
- Develop a comprehensive training program for service providers, including a Training of Trainers (ToT) module, pre-and post-training assessments, feedback collection, and certification processes.
- Conduct a competitive selection process for NGO representatives participating in the training program. Up to three service providers per regional sub-grantee will be trained based on professional experience and organizational role.
- Deliver at least two offline training sessions for 17 NGOs, equipping service providers with structured skills for PSS delivery.

**2.3 Supervision and Support Mechanisms**

- Provide six months of structured supervision for trained NGOs, including individual and group sessions (minimum one hour per participant per month).
- Establish a Community of Practice to facilitate peer exchange, best practice sharing, and discussion of implementation challenges.
- Offer ongoing expert and technical assistance to ensure fidelity to the intervention model.

**2.4 Implementation and Service Delivery**

- NGOs will provide individual and group counseling sessions incorporating self-help techniques and evidence-based PSS strategies.
- NGOs will integrate structured mental health support into their routine service delivery to ensure sustainability.
- Special attention will be given to developing internal policies on PSS provision and ensuring structured referral pathways for specialized mental health services.

**2.5 Monitoring, Evaluation, and Reporting**

- Develop a data collection framework and key performance indicators (KPIs) to assess service quality and effectiveness.
- Collect and analyze training assessment data, feedback forms, and service delivery reports.
- NGOs will systematically document the number of beneficiaries reached, service quality improvements, and lessons learned.
- Final project outcomes will be synthesized into a report assessing intervention effectiveness and long-term sustainability.

1. **Expected Outcomes**

- Capacity building of NGO personnel: Service providers gain structured skills in delivering psychosocial support.
- Sustainable service integration: NGOs institutionalize structured PSS interventions into their operations.
- Increased access to mental health support: Beneficiaries, including PLHIV, key populations, and NGO personnel, receive targeted psychosocial services.
- Improved resilience and well-being: Strengthened community-based support mechanisms contribute to mental health improvements.
